# Our Journey from Individual Efforts to Nationwide Support: Implementing Newborn Screening for Spinal Muscular Atrophy in Serbia

**DOI:** 10.3390/ijns10030057

**Published:** 2024-08-15

**Authors:** Miloš Brkušanin, Nemanja Garai, Jelena Karanović, Tamara Šljivančanin Jakovljević, Aleksandra Dimitrijević, Kristina Jovanović, Tanja Lazić Mitrović, Željko Miković, Goran Brajušković, Dimitrije Mihailo Nikolić, Dušanka Savić-Pavićević

**Affiliations:** 1 Centre for Human Molecular Genetics, Faculty of Biology, University of Belgrade, 11000 Belgrade, Serbia; nemanja.garai@bio.bg.ac.rs (N.G.); jkaranovic@bio.bg.ac.rs (J.K.); brajuskovic@bio.bg.ac.rs (G.B.); 2 Department of Neonatology, Obstetrics and Gynaecology Clinic Narodni Front, 11000 Belgrade, Serbia; tamaricasljiva@hotmail.com (T.Š.J.); tanjalm01@gmail.com (T.L.M.); 3 Department of Gynecology and Obstetrics, Faculty of Medical Sciences, University of Kragujevac, 34000 Kragujevac, Serbia; saskadkg@gmail.com; 4 Gynaecology and Obstetrics Clinic, Clinical Centre of Kragujevac, 34000 Kragujevac, Serbia; 5 Neurology Department, University Children’s Hospital, 11000 Belgrade, Serbia; kristina.jovanovic104@gmail.com (K.J.); dimitrije.nikolic@gmail.com (D.M.N.); 6 High-Risk Pregnancy Unit, Obstetrics and Gynaecology Clinic Narodni Front, 11000 Belgrade, Serbia; mikovic.zeljko@gmail.com; 7 Faculty of Medicine, University of Belgrade, 11000 Belgrade, Serbia

**Keywords:** spinal muscular atrophy, newborn screening, neonatal screening, population screening, feasibility study, genetic testing, public health, real-time PCR, SMN1, motor neuron disease

## Abstract

Innovative treatments for spinal muscular atrophy (SMA) yield the utmost advantages only within the presymptomatic phase, underlining the significance of newborn screening (NBS). We aimed to establish statewide NBS for SMA in Serbia. Our stepwise implementation process involved technical validation of a screening assay, collaboration with patient organizations and medical professionals, a feasibility study, and negotiation with public health representatives. Over 12,000 newborns were tested during the 17-month feasibility study, revealing two unrelated SMA infants and one older sibling. All three children received therapeutic interventions during the presymptomatic phase and have shown no signs of SMA. No false-negative results were found among the negative test results. As frontrunners in this field in Serbia, we established screening and diagnostic algorithms and follow-up protocols and raised awareness among stakeholders about the importance of early disease detection, leading to the incorporation of NBS for SMA into the national program on 15 September 2023. Since then, 54,393 newborns have been tested, identifying six SMA cases and enabling timely treatment. Our study demonstrates that effective collaborations between academia, non-profit organizations, and industry are crucial in bringing innovative healthcare initiatives to fruition, and highlights the potential of NBS to revolutionize healthcare outcomes for presymptomatic SMA infants and their families.

## 1. Introduction

Spinal muscular atrophy (SMA) constituted the predominant genetic etiology underlying infant mortality during the period preceding the advent of groundbreaking genetically engineered therapeutic interventions [1]. This devastating, autosomal-recessive disorder is caused by functional loss of the survival of motor neuron (SMN) protein necessary for survival and normal function of motor neurons. Clinical diagnosis of SMA has to be confirmed by genetic testing. In ~95–98% of affected individuals, functional loss of the SMN protein is due to the homozygous absence of the *SMN1* gene (deletion or gene conversion into *SMN2*) (OMIM# 600354) [2]. The remaining ~2–5% of affected individuals are compound heterozygotes, showing the absence of one *SMN1* allele and a small mutation (point mutation, small deletion, or small insertion) in the other allele [3]. In humans, a nearly identical copy of the *SMN1* gene is known as *SMN2* (OMIM# 601627). Due to a synonymous variant in exon 7 (c.840C>T, NM_000344.4), only ~10% of *SMN2* mRNA is correctly spliced and produces a full-length transcript [4]. Hence, *SMN2* is the only source of the functional SMN protein in SMA individuals, which makes it a major genetic modifier of SMA and a target of two innovative therapies [5,6,7,8,9].

The breakthrough in SMA therapy came with the approval of an antisense oligonucleotide Nusinersen (Spinraza^®^, Biogen, Cambridge, MA, USA) targeting *SMN2* [8], followed by the *SMN1* gene replacement therapy (Zolgensma^®^, AveXis, Inc., Basel, Switzerland) [10] and a small molecule for correction of the *SMN2* splicing defect (Evrysdi™, Genentech USA, Inc., South San Francisco, CA, USA) [9]. Independently of the therapeutic approach, clinical and preclinical data, as well as real-world data, demonstrate that early postnatal induction of SMN, before the irreversible neuronal loss, is critical for the best response and prevention of major disability [11,12,13]. However, significant diagnostic delays are common in SMA, with delays of ~2 months in type 1, ~5 months in type 2, and ~17 months in type 3 patients [14,15]. Consequently, many patients have already passed the narrow and critical therapeutic time-window when the maximal protective effect is achievable. Therefore, implementing mandatory nationwide newborn screening (NBS) for SMA has become an essential, global, and urgent objective in the SMA field to ensure timely diagnosis and intervention, maximizing the potential for positive clinical outcomes.

Serbia has a longstanding tradition and substantial expertise in clinical and molecular-genetic research into SMA, dating back to 1997 when the Faculty of Biology in Belgrade established molecular-genetic testing for the disease. Since 2011, our institution has validated and successfully employed multiplex ligation-dependent amplification (MLPA), specifically the SALSA MLPA Probemix P021 SMA (MRC Holland, Amsterdam, The Netherlands), for SMA diagnosis, reconstruction of complex genetic rearrangements within the SMA region, including hybrid *SMN* copies, and determination of the *SMN2* copy number (7). Together with the Institute for Mother and Child Health Care of Serbia “Dr. Vukan Cupic”, we have enabled definitive diagnosis in hundreds of individuals with clinical symptoms of SMA, as well as genetic counseling for affected families and prevention of the disease in at-risk families through carrier and prenatal testing. However, the introduction of Nusinersen and Risdiplam in Serbia in July 2018 represented a significant milestone in the management of SMA. Provided by the Republic Health Insurance Fund, these therapies were administered to symptomatic individuals, which significantly hindered the achievement of maximum therapeutic efficacy. The availability of these treatments and the emphasis on timely intervention have led to a paradigm shift in the approach to SMA prevention. Newborn screening has emerged as the sole ethically justifiable strategy for preventing major disability in SMA and for achieving maximum outcomes, ensuring universal access for every infant and family, and rational allocation of our healthcare system’s limited financial resources in the long term. Consequently, it became an essential and logical next step to pursue.

This paper chronicles our journey, detailing the incremental steps and challenges that culminated in the implementation of statewide NBS for SMA in Serbia. We hope it can serve as a guide for other countries looking to introduce newborn screening for SMA, providing practical advice on how to navigate the complexities of such a significant public health initiative.

## 2. Materials and Methods

### 2.1. Validation Study of a Newborn Screening Assay

In 2018, we conducted the technical validation of a commercial assay for NBS for SMA at the Faculty of Biology in Belgrade (SALSA MC002 SMA Newborn Screen Assay, MRC-Holland, Amsterdam, The Netherlands). The assay utilizes PCR and melt curve analysis to detect the presence or absence of the *SMN1* and *SMN2* genes in DNA extracted from dried blood spots (DBS) and is described in detail in Strunk et al. [16]. A total of 96 carefully chosen samples with varying *SMN1* and *SMN2* copy numbers, previously determined by MLPA, were used for assay validation. Homemade DBS were prepared by pipetting 50 µL of whole blood onto the filter paper NUCLEIC-CARD™ matrix, 4 spots (Thermo Fisher Scientific, Waltham, MA, USA) and dried overnight at room temperature. The crude DNA extraction from a washed ~3.2 mm diameter punch was conducted according to the MC002 Instructions for Use [17]. Analysts were blinded to the expected results, and approximately 30% of randomly selected samples were tested in duplicate to ensure accuracy. A sample was considered SMA-positive if the *SMN1*-peak was absent and the *SMN2*-peak was present. Conversely, if the *SMN2*-peak was absent and the *SMN1*-peak was present, this completely excluded SMA diagnosis. This is due to the fact that SMA individuals with no (functional) *SMN1* gene always have at least one copy of *SMN2*, as the complete absence of *SMN1* and *SMN2*, and consequently of the SMN protein, results in embryonic lethality [18,19]. If both *SMN1*- and *SMN2*-peak are absent, or if the DNA quantity fragment (Q-fragment) melt peak at 49 °C is the highest peak present, the reaction should be repeated.

### 2.2. Collaborative Efforts Leading to Feasibility Study of the Newborn Screening for SMA

In 2021, our research team collaborated closely with the Association SMA Serbia and the Neurology Department of the University Children’s Hospital to design a feasibility study on NBS for SMA in Serbia. Our project proposal was rejected by the Ministry of Health, underscoring the challenges of securing funding and support for innovative healthcare initiatives. However, this setback did not deter us from pursuing our goal. The Association SMA Serbia took the initiative to organize a roundtable discussion on SMA in 2021, which brought together representatives from pharmaceutical companies who recognized the pressing need to screen for this life-altering condition. Notably, these industry partners demonstrated their commitment to supporting this vital cause by providing complete financial backing for a one-year feasibility study.

### 2.3. The Feasibility Study of the NBS for SMA in Serbia

The main objective of the feasibility study of the NBS for SMA in Serbia was to prepare the ground for the establishment of the national NBS for this disease through organized and coordinated work at different levels of health care—from collection and transport of dried blood spot samples, through laboratory testing, timely reporting of results to pediatricians, informing parents, to swift therapy approval and application, and pathways of patient care and monitoring. The study objective was in line with the improvement of the availability of newborn screening and treatment of rare diseases addressed by the “Rare Diseases Program in the Republic of Serbia for the period 2020–2022” [20]. For this purpose, and bearing in mind the average incidence of the disease [21], the largest maternity hospital in the country was chosen, namely the Obstetrics and Gynaecology Clinic Narodni Front in Belgrade, in which around 8000 children are born annually. This study was conducted in accordance with the Declaration of Helsinki and approved by the Ethics Committee of the Obstetrics and Gynecology Clinic Narodni Front in Belgrade (Ethics Approval number 05006-2022-6579 from 11 April 2022). The study was officially launched on 12 April 2022. We employed an informed consent model, where mothers were fully informed about NBS before providing written consent. This approach granted parents decision-making authority and allowed them to “opt in” to the screening. Previous fundraising campaigns for gene therapy have raised awareness in Serbia about the burden of SMA and the critical significance of early intervention. To continuously promote the study, project participants made numerous media appearances, encouraging enrollment into the program.

To prevent potential sample contamination during sample collection, laboratory gloves and masks were mandated for medical staff during sampling to avoid contact with bare hands or talking above samples without protection. Furthermore, in collaboration with the Croatian team responsible for implementing SMA NBS in Croatia, we developed a large-format poster with step-by-step instructions and accompanying pictures to facilitate the sampling process for nursing staff. This approach ensured a standardized and contamination-free sample collection protocol.

During the feasibility study, a separate Guthrie card adjusted for genetic analyses was used (NUCLEIC-CARD™ matrix, 1 spot, Thermo Fisher Scientific, USA), but the blood sampling was performed at the same time (between 48 and 72 h of life), and from the same heel prick as for the mandatory screening for other conditions. Even though we successfully tested the standardized Whatman 903 filter paper card (Eastern Business Forms, Inc., Mauldin, SC, USA), which is commonly used in routine newborn screenings in Serbia, we opted for the NUCLEIC-CARD matrix for genetic analysis throughout the feasibility study in order to ensure the best sample quality, preservation, and long-term storage of nucleic acids. In cases where the neonate’s condition was critical and required immediate transportation to another institution or transfusion, we advised that sampling for SMA screening be conducted immediately prior to transportation. This approach was implemented to minimize delays. Screening for SMA was designed as a standalone genetic test based on the previously validated PCR and melt curve analysis using QuantStudio™ 5 Real-Time PCR System (Applied Biosystems by Thermo Fisher Scientific).

After completing the initial year of our study, it became evident that incorporating SMA screening as a routine part of the national NBS program necessitated additional time. Consequently, we opted to extend and expand the study by incorporating an additional maternity hospital outside of Belgrade, specifically the Gynecology and Obstetrics Clinic at the Clinical Centre of Kragujevac (Ethics Approval number 01–23/166 from 9 May 2023). This expansion allowed us to broaden our reach to newborns beyond the capital city, increasing the number of screened newborns by up to 30% compared to the first year, and enabling more accurate estimation of turnaround time. Furthermore, we implemented sample shipping by mail as a mode of transport, optimized the care pathway for families residing outside the capital, and introduced sample barcoding in our laboratory. These enhancements represented significant improvements compared to concurrent screenings. Moreover, we continued negotiations with the Institute of Public Health of Serbia “Dr. Milan Jovanović Batut” and the Republic Health Insurance Fund, presenting a strategy for addressing logistical, infrastructural, and financial challenges related to screening expansion, as well as emphasizing the urgency of transitioning to a state-wide program and the ethical implications of not screening newborns nationwide.

## 3. Results

### 3.1. Results of the Validation Study of a NBS Assay

The results of our validation study of a NBS assay (SALSA MC002 SMA Newborn Screen Assay, MRC-Holland, The Netherlands) demonstrated that the method exhibited 100% specificity and sensitivity in identifying individuals with SMA who had the most common underlying genetic defect—homozygous absence of the *SMN1* gene, enabling identification of ~95–98% of SMA individuals. Consequently, the overall specificity and sensitivity of the assay in diagnosing SMA is 95–98%. The only exception is the absence of the *SMN2* signal, which completely excludes SMA diagnosis. The assay was unable to detect SMA carriers or determine *SMN1* and *SMN2* copy numbers. Furthermore, it was not capable of detecting compound heterozygotes, which account for approximately 2–5% of SMA cases. No false-negative or false-positive results were obtained during the validation study. The assay’s workflow took approximately 3 h to process 92 samples, from sample preparation to result generation. This validation process using samples with known *SMN1* gene status ensured that the assay was reliable and accurate, providing a solid foundation for the next step—a feasibility study conducted on a limited territory and timeframe.

### 3.2. Results of the Feasibility Study of the NBS for SMA in Serbia

The feasibility study lasted 17 months, during which over 12,000 newborns underwent testing. SMA diagnosis was confirmed in two unrelated newborns (four *SMN2* copies and three *SMN2* copies) from families with no prior history of SMA. The average time between birth and the first screen-positive result was 4 days, with an additional 2 days required for final confirmation of the diagnosis. Following diagnosis, therapy approval was obtained within 10 days, and treatment with Risdiplam was initiated at the age of 17 and 25 days, respectively. At the time of diagnosis, Risdiplam was administered off-label, as it was not yet approved by the European Medicines Agency for use in infants under 2 months of age. However, we were guided by the U.S. Food and Drug Administration’s approval of Risdiplam for all ages [22]. Additionally, an older sibling of one of the infants, who was 16 months old at the time, was also diagnosed with SMA (four *SMN2* copies) during the clinically silent phase and immediately began therapy with Nusinersen. All three children achieved maximum scores on functional tests specific to their ages before treatment initiation. The screening assay revealed that 10% of the tested newborns had the *SMN1* gene, but lacked the *SMN2* gene, enabling 100% exclusion of SMA diagnosis. Importantly, there were no false positives from the initial DBS testing, and no false-negative results have been reported to date among newborns who tested negative during screening. The feasibility study provided timely access to available treatments and genetic counseling, thereby minimizing harm and maximizing benefits for SMA-positive newborns and their families.

**Figure 1:**
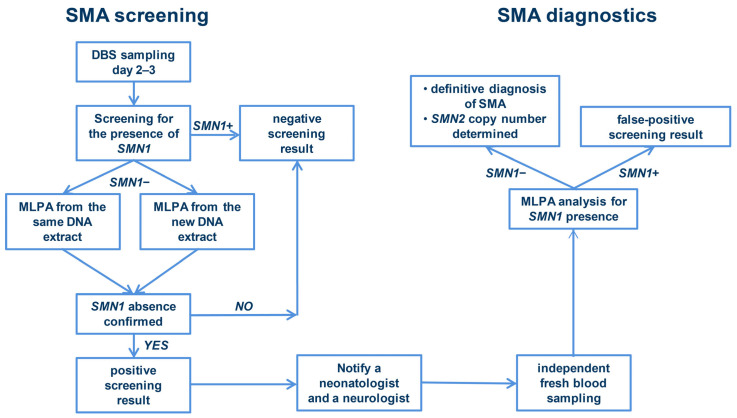
Schematic representation of the screening and diagnostic algorithm for spinal muscular atrophy in Serbia. SMA: spinal muscular atrophy; DBS: dried blood spot; *SMN1*+: the *SMN1* gene is present; *SMN1*−: the *SMN1* gene is absent; MLPA: multiplex ligation-dependent probe amplification. SALSA MLPA Probemix P021 SMA (MRC Holland, Amsterdam, The Netherlands) is used in all MLPA reactions.

During the feasibility study, we developed and implemented a screening and diagnostic algorithm (Figure 1), which enabled the successful identification of SMA-positive newborns. The algorithm consists of a screening phase, followed by a diagnostic phase. The screening phase consisted of an initial screening step and, in case of a positive result, MLPA was performed twice: once using DNA extract from the initial screening sample and once using an additional punch prepared from the same Guthrie card. The SALSA MLPA Probemix P021 SMA diagnostic kit (MRC Holland, Amsterdam, The Netherlands) was employed for this purpose. The rapid performance of MLPA allowed for immediate confirmation of the positive screening result and quantification of the *SMN2* copy number prior to the completion of the screening phase. The triple-positive results obtained from two independent punches of the same dried blood spot further ensured that this particular DBS was indeed positive and that there were no sample mix-ups during the punching step or reaction preparation. This approach enabled a high degree of confidence in the overall accuracy of the results, thereby ensuring that SMA-positive newborns were accurately identified.

In cases where all three DBS tests with two independent methods yielded positive results, we proceeded to the diagnostic phase of the algorithm and implemented the corresponding patient care pathway. Firstly, we notified the study coordinator at the maternity hospital, who provided us with the family’s contact information. We subsequently communicated the screening result (including the *SMN2* copy number) and family contact to a pediatric neurologist at the University Children’s Hospital. The neurologist promptly contacted the family and scheduled a confirmatory blood sampling and clinical evaluation within 24 h. Fresh blood samples were collected for the definitive confirmation of the screening result, precise determination of *SMN2* copy number, and potential biomarker testing at baseline. Each confirmed SMA case was reported to the Association SMA Serbia and documented in their local registry, as no national registry for SMA has yet been established in Serbia. Treatment options were discussed with the family based on the *SMN2* copy number, bearing in mind that during the feasibility study, only *SMN2*-splicing modifiers were available. Regardless of treatment choice, we insisted on immediate treatment for all SMA-positive newborns, including those with four *SMN2* copies. Long-term follow-up of SMA-positive newborns required regular visits to the neuromuscular team (intervals depended on the therapy), when an additional blood sample was taken for biomarker identification.

During the last five months of the feasibility study, we included an additional maternity hospital located outside of the capital of Serbia. Our efforts did not cease there. We implemented a public awareness campaign to highlight the critical importance of early SMA detection and disseminated the project’s main message to the public and government officials, seeking support for the expansion of screening across the entire territory of Serbia, where approximately 64,225 children are born annually (based on a decade-long analysis of birth data from 2013–2022) [23]. We managed to alleviate the emotional burden faced by parents upon receiving an unexpected diagnosis by expediting the approval process for therapy for all affected infants, including those with four copies of the *SMN2* gene. Through these activities, we successfully increased parental consent for study participation. Initially, the refusal rate among mothers was around 5%. However, by the end of the project, this rate decreased to less than 2%, indicating a significant improvement in parental willingness to participate in the screening process.

### 3.3. Key Findings of the National NBS for SMA in Serbia

**Figure 2:**
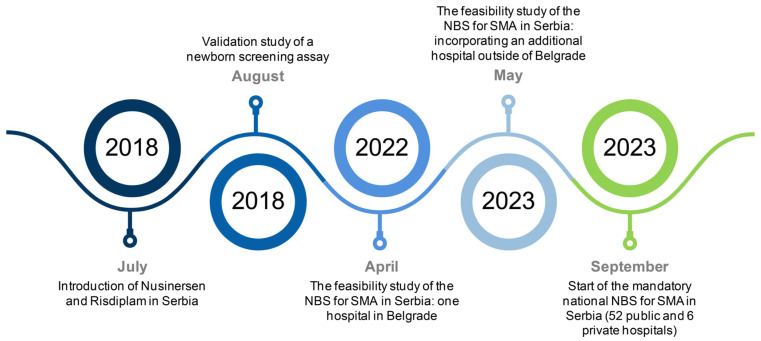
A timeline of the key milestones leading to the implementation of mandatory statewide newborn screening for spinal muscular atrophy in Serbia. The timeline does not include collaborative efforts and negotiations with governmental bodies. NBS: newborn screening; SMA: spinal muscular atrophy.

Following the success of our feasibility study, we collaborated with the Republic Health Insurance Fund and the Institute of Public Health of Serbia “Dr. Milan Jovanović Batut” to establish a nationwide NBS program for SMA in Serbia. Even though we did not receive support from the Ministry of Health, we tirelessly and successfully negotiated with the Government of the Republic of Serbia to designate the Faculty of Biology, University of Belgrade as the reference national institution for this initiative on 6 September 2023. We have since significantly transformed our approach to preventing SMA in Serbia. As of 15 September 2023, we have implemented a statewide mandatory NBS program that covers all newborns. The program involves collecting dried blood spots from 52 public and 6 private maternity hospitals from the entire country on a separate and standardized Whatman 903 filter paper card. The cards are shipped to the Faculty of Biology in Belgrade using postal services. Here, they undergo genetic screening utilizing the abovementioned algorithms. Figure 2 illustrates a timeline of the significant milestones that culminated in the establishment of mandatory statewide NBS for SMA in Serbia. In the meantime, we also validated and started using an additional set of reagents based on real-time PCR (Targeted qPCR SMA FLEX, LaCAR MDx Technologies/ZenTech, Liege, Belgium) and are currently using both Targeted qPCR SMA FLEX and SALSA MC002 SMA Newborn Screen (MRC Holland, Amsterdam, The Netherlands) in the national newborn screening program. Immediately before the approval of the national NBS for SMA, gene therapy was registered in our country. Considering the limited treatment budget and the need to prioritize access to gene therapy, the Republic Expert Commission for SMA was established with the aim of defining clear criteria for an optimal treatment approach based on comprehensive clinical and genetic information for each SMA newborn. As NBS for SMA has become mandatory in Serbia, informed consent from parents is no longer required. However, parents retain the right to opt out of NBS and must sign a statement of refusal in such cases. To date, our national screening program has encompassed 54,393 newborns, resulting in the identification and treatment of six SMA-positive newborns (two with two *SMN2* copies, three with three *SMN2* copies, and one with four *SMN2* copies). All identified newborns had a homozygous absence of the entire *SMN1* gene, with no hybrid *SMN* copies observed. The decision regarding therapy is made in consensus between expert advice and parental wishes. Large-scale newborn screening for SMA not only provides timely diagnosis and prompt initiation of therapy, but also offers valuable epidemiological insights, such as the estimation of SMA incidence. Based on the results of our national screening program, we estimate the current incidence of SMA in Serbia to be 1:9066, which aligns with incidence data from other European countries [21]. As the program continues to expand, we anticipate achieving more accurate incidence estimates in Serbia. Our team is currently working to expand the list of disorders that can be genetically screened for through NBS.

## 4. Discussion

Newborn screening for SMA has been recognized as a crucial public health strategy to prevent irreversible neurological damage and improve patient outcomes [24]. In the United States, from the start of 2024, all newborns are screened for SMA at birth [25]. In contrast, only 58% of newborns in geographical Europe are tested at birth, as reported by the SMA Newborn Screening Alliance interactive map of Europe [26]. Our interdisciplinary team, consisting of experts in molecular genetics, pediatric neurology, neonatology, and patient advocacy, pursued a vision to provide optimal benefits to SMA individuals and their families in Serbia. Through a stepwise implementation process, we successfully established a statewide NBS for SMA in Serbia. This process consisted of three distinct phases: (1) laboratory validation of the NBS assay using samples with known *SMN1* status to ensure accuracy and reliability; (2) a feasibility study on a smaller cohort of newborns to assess the effectiveness and practicality of the screening protocol; and (3) transition to a national program, where we further refined the screening process.

Multiple methods are available for the NBS of SMA, which can target either the *SMN1* and *SMN2* genes or *SMN1* and a reference gene, or even test multiple diseases simultaneously. All these methods enable rapid and precise genetic screening of samples and should be internally validated in the screening laboratory. Among the available methods, our preferred approach is one that simultaneously targets both the *SMN1* and *SMN2* genes, because in the absence of the *SMN2* signal this approach ensures exclusion of SMA diagnosis with 100% certainty. As we mentioned above, this is because individuals lacking functional *SMN1* would be expected to exhibit embryonic lethality in the absence of functional *SMN2* [18,19]. The frequency of homozygous absence of the *SMN2* gene in the Serbian population appears consistent with other reports [27]. However, when both *SMN1* and *SMN2* genes or *SMN1* and a reference gene are present, the certainty of a negative screening result is 95–98%. This may lead to false negative results due to compound heterozygotes, who possess one non-functional *SMN1* allele, and will therefore be missed by the screening methods. Based on our extensive experience, the most common point mutation present in >90% of SMA compound heterozygotes in Serbia is a missense mutation c.821C>T (NM_000344.4) (p.Thr274Ile) in exon 6. This mutation is always found in combination with 1 or 2 *SMN2* copies, resulting in type 2 or type 3 SMA, respectively [28]. Although these individuals will give a false negative screening result and only be discovered as symptomatic, their clinical phenotype is expected to be milder than that of those with a complete absence of *SMN1* and the same number of *SMN2* copies. Current screening assays are also unable to identify carriers of one *SMN1* copy. Nevertheless, even diagnostic methods such as MLPA have limitations, failing to detect carriers with two copies of *SMN1* on the same chromosome (the “2 + 0” genotype) or carriers of small mutations [29]. This highlights the importance of acknowledging the limitations of current screening approaches. It is crucial to inform stakeholders and the general public about these limitations to ensure accurate understanding and management of SMA and to create realistic expectations.

During the feasibility study, we created the algorithm for successful screening and diagnosis of SMA. An important part of this algorithm is performing MLPA during the screening phase, particularly determining the *SMN2* copy number at the end of the screening phase. The *SMN2* copy number is crucial for clinicians before they inform the parents of a positive screening result, as it allows them to adjust their communication with parents at the patient’s first visit regarding the urgency of the case and potential therapeutic options available for the child. To ensure the reliability of *SMN2* copy number determination, we perform this analysis twice given that small amounts of DNA extracted from dried blood spots may lead to less accurate results. To ensure timely diagnosis and effective management, it is essential that both the screening and diagnostic phases are conducted in a single laboratory, allowing for the rapid establishment of a definitive diagnosis. It is imperative to highlight the specialized nature of NBS for SMA, which requires expertise in genetic testing, familiarity with SMA genetics, and proficiency in verifying screening results. Our experience and that of surrounding countries demonstrate that governments or policymakers often underestimate the complexity of this task, assuming that any laboratory can perform it. However, in reality, while many laboratories have personnel and instrumentation capable of conducting high-throughput testing, they may lack the necessary expertise to interpret or confirm results. Consequently, it is crucial that SMA NBS screening is performed in a laboratory with specific expertise and resources to guarantee accurate, reliable, and timely results.

In our study, we strongly encouraged immediate therapy approval even for newborns with 4 *SMN2* copies. This approach was based on the treatment algorithm developed by a multidisciplinary team of expert clinicians and scientists in the field of SMA [30], but also on our previous findings on phosphorylated neurofilament heavy chain levels (pNF-H). Our research has shown that clinically silent infants with SMA carrying three or four *SMN2* copies can display elevated pNF-H levels in cerebrospinal fluid and plasma, indicating early neuronal degeneration [31]. Based on this result, we established a follow-up protocol for newly diagnosed SMA cases, which includes: (1) baseline assessment of motor functions using age-appropriate scales; (2) follow-up assessments of motor functions after one year of treatment; and (3) longitudinal measurement of cerebrospinal fluid and plasma pNF-H levels (cerebrospinal fluid measurements are only conducted for individuals treated with Nusinersen). This protocol aims to monitor treatment response and potential complications, thereby guiding future decision-making.

In this transformative journey, we transitioned from being isolated individuals and visionaries who championed a singular idea to an entire community and nation that now acknowledges the paramount significance of newborn screening. Our experience highlights the value of collaboration between academia, healthcare professionals, patient advocates, and policymakers in developing and implementing effective NBS programs.

## 5. Conclusions

Our stepwise approach enabled us to overcome challenges, secure public and government support, and facilitate rapid statewide implementation of SMA NBS. We hope that the successful implementation of SMA NBS in Serbia may serve as a model for other countries seeking to establish similar programs. We have proved that, by early detection and intervention, we can improve patient outcomes and reduce the burden of SMA on affected individuals and their families. However, with the accessibility of treatment options and NBS for SMA, new challenges arise in selecting the most appropriate treatment and establishing effective follow-up protocols for patients undergoing treatment.

## Data Availability

The data are not publicly available due to privacy restrictions.
